# Association between Lipid Levels, Anti-SARS-CoV-2 Spike Antibodies and COVID-19 Mortality: A Prospective Cohort Study

**DOI:** 10.3390/jcm12155068

**Published:** 2023-08-01

**Authors:** Sylvia Mink, Christoph H. Saely, Matthias Frick, Andreas Leiherer, Heinz Drexel, Peter Fraunberger

**Affiliations:** 1 Central Medical Laboratories, 6800 Feldkirch, Austria; 2 Medical-Scientific Faculty, Private University of the Principality of Liechtenstein, 9495 Triesen, Liechtenstein; 3 VIVIT Institute, Academic Teaching Hospital Feldkirch, 6800 Feldkirch, Austria; 4 Department of Internal Medicine, Academic Teaching Hospital Feldkirch, 6800 Feldkirch, Austria; 5 Drexel University College of Medicine, Philadelphia, PA 19129, USA

**Keywords:** SARS-CoV-2, LDL-C, total cholesterol, HDL-C, triglycerides, lipid profile, anti-SARS-CoV-2 antibodies, in-hospital mortality, COVID-19

## Abstract

Background: Recent studies suggest that both lipid levels and anti-severe-acute-respiratory-syndrome-coronavirus-2 (SARS-CoV-2) antibody levels are associated with outcome in coronavirus disease 2019 (COVID-19). While both parameters have separately been implicated in the neutralization and clearance of pathogens during severe infections, it is currently unclear whether the interplay of these parameters affects outcome in COVID-19. We therefore aimed to determine whether there was a relationship between lipoproteins, anti-SARS-CoV-2 antibodies, and COVID-19 mortality. Methods: In this prospective, multicenter cohort study, we recruited 1152 hospitalized patients with COVID-19 from five hospitals. Total cholesterol (TC), LDL-C, HDL-C, triglycerides, and anti-SARS-CoV-2 spike antibodies were measured on hospital admission. The investigated endpoint was in-hospital mortality. Results: LDL-C, HDL-C, and TC were significantly lower in non-survivors than in survivors (mg/dL, 95%CI; 56.1, 50.4–61.8 vs. 72.6, 70.2–75.0, *p* < 0.001; 34.2, 31.7–36.8 vs. 38.1, 37.2–39.1, *p* = 0.025; 139.3, 130.9–147.7 vs. 157.4, 54.1–160.6, *p* = 0.002). Mortality risk increased progressively with lower levels of LDL-C, HDL-C, and TC (aOR 1.73, 1.30–2.31, *p* < 0.001; 1.44, 1.10–1.88, *p* = 0.008; 1.49, 1.14–1.94, *p* < 0.001). Mortality rates varied between 2.1% for high levels of both LDL-C and anti-SARS-CoV-2 antibodies and 16.3% for low levels of LDL-C and anti-SARS-CoV-2 antibodies (aOR 9.14, 95%CI 3.17–26.34, *p* < 0.001). Accordingly, for total cholesterol and anti-SARS-CoV-2 antibodies, mortality rates varied between 2.1% and 15.0% (aOR 8.01, 95%CI 2.77–23.18, *p* < 0.001). Conclusion: The combination of serum lipid levels and anti-SARS-CoV-2 antibodies is strongly associated with in-hospital mortality of patients with COVID-19. Patients with low levels of LDL-C and total cholesterol combined with low levels of anti-SARS-CoV-2 antibodies exhibited the highest mortality rates.

## 1. Introduction

Previous studies have suggested that plasma lipoproteins are vital for the removal of pathogen-associated lipids and toxins in severe infections such as sepsis [1,2,3,4]. Although the pathophysiological role of lipoproteins in COVID-19 is still incompletely understood, experimental studies show that the lipid biogenesis pathways of the host may affect virus replication in several ways [5]. Viral internalization into a cell requires the attachment and fusion of viral membrane with plasma membrane by way of endocytosis [6,7]. Lipids can act as direct receptors or as entry co-factors at both the cell surface and the endosomes [8,9]. In experimental studies, enhanced transport of cholesterol from the cell membrane to the endoplasmatic reticulum by the enzyme cholesterol-25-hydroxylase inhibits SARS-CoV-2 entry into a cell by depriving it of accessible cholesterol at the plasma membrane. Conversely, this entry inhibition can be removed by adding soluble cholesterol [10]. Lipids further play an important role in the formation and function of the viral replication complex [11,12]. Accordingly, in animal models, intracellular cholesterol was found to enhance SARS-CoV2 infectivity [13,14].

In clinical studies, COVID-19 is associated with increased rates of dyslipidaemia, which may persist up to one year after infection [15,16]. Pre-existing dyslipidaemia and atherosclerosis have been linked to increased severity and mortality of COVID-19 [17,18]. In addition, several studies describe an association between plasma lipid levels and outcome in patients infected with SARS-CoV-2, suggesting that plasma lipids may be involved in the immune response to COVID-19 [19,20,21,22].

Accordingly, antibodies are known to play a crucial role in the neutralization of pathogens. With regard to COVID-19, neutralizing antibodies have been correlated with lower infection rates and protection against severe courses of COVID-19 [23,24,25,26]. Positive results have been reported following the administration of neutralizing monoclonal antibodies [27,28,29]. In addition, previous studies have demonstrated a protective effect of booster vaccinations against severe illness and hospitalization due to COVID-19 [30,31,32,33,34], and high anti-SARS-CoV-2 spike antibody levels on hospital admission have been shown to be associated with lower in-hospital mortality [35].

Taken together, these points suggest that both lipoproteins and antibodies are of high importance in the immune response against SARS-CoV-2. However, it is currently unclear whether the interplay between lipoproteins and antibodies affects outcome in SARS-CoV-2 infections. We therefore evaluated whether there was a relationship between anti-SARS-CoV-2 spike antibody, lipoprotein levels and COVID-19 mortality.

## 2. Methods

### 2.1. Study Design and Participants

The design of this study has previously been reported elsewhere [35]. In brief, we conducted a prospective, multicenter cohort study in which we enrolled patients from five Austrian hospitals who were hospitalized between 1 August 2021 and 10 April 2022.

Patients were considered eligible for recruitment if they tested positive for SARS-CoV-2 with a polymerase chain reaction (PCR)-based assay and a blood sample was drawn on hospital admission. Patients were excluded from the study if no blood sample was collected, if the residual sample material was insufficient, if they had been previously hospitalized during the study period, or if their hospital stay had not concluded by the end of the study.

Sample size calculation for logistic regression with a binary covariate, Wald’s method, yielded a minimum sample size of 465 participants (alpha 0.05, two-sided, power 0.8).

### 2.2. Variables

The investigated endpoint was in-hospital mortality from any cause.

Diabetes status was defined based on previous diagnosis according to ICD-10 codes or HbA1c levels above 6.4%, as obtained from patient files. Patients with a BMI of 30 or above were classified as obese.

Known risk factors for severe courses and mortality in COVID-19 were chosen as predefined covariates. It has been well documented that obesity and an underlying condition of diabetes predispose to a higher mortality risk through a combination of inflammation, hypercoagulation, and mechanical obstruction [36]. Age, due to the accumulation of underlying conditions and frailty, is another known risk factor for severe course and mortality in COVID-19 [37]. Statin therapy was chosen as an additional covariate because of its effects on lipid levels.

Laboratory parameters that were used as covariates were anti-SARS-CoV-2 antibodies, total cholesterol, LDL-C, HDL-C, and triglyceride levels.

### 2.3. Data Sources and Measurements

Total cholesterol, LDL-C, HDL-C, and triglycerides were measured on hospital admission using the respective Roche assays on Roche Cobas 6000 or Cobas 8000 systems. Lipid measurements were conducted with the Roche CHOL2 assay for cholesterol, the HDL-C4 assay for HDL-C, the LDL-C3 assay for LDL-C, and the TRIGL assay for triglycerides. The Roche LDL-C3 assay allows direct measurement of LDL-C utilizing a chromogenic enzymatic method. Anti-SARS-CoV-2 spike antibodies were measured using the Elecsys Anti-SARS-CoV-2 S assay.

Clinical data obtained from patient files included age, gender, body mass index (BMI), vaccination status, type of vaccine, COVID-19 variant, PCR-derived cycle threshold values, reason for hospitalization, main diagnosis, symptoms at admission such as coughing, fever, loss of taste and/or smell, oxygen requirement (none/without endotracheal intubation/with endotracheal intubation), duration of hospital stay, and, if applicable, duration in intensive care unit (ICU) as well as in-hospital survival.

### 2.4. Statistical Methods

All statistical analyses were calculated using the IBM Statistical Package for the Social Sciences (SPSS), version 28 (IBM, Armonk, NY, USA). Baseline statistical characteristics, including frequencies, percentages, means, medians, standard deviations, and interquartile ranges were determined with the functiondescriptive statistics. The 95% confidence intervals for means were calculated with one-sample *t* tests. Statistical significance was determined by Mann–Whitney *U*, chi-square test, or log-rank test, as appropriate. As is customary, two-sided *p* values of <0.05 were considered statistically significant.

In order to quantify the respective risk associated with lower lipid levels, we built logistic regression models. Odds ratios with a 95% confidence interval were calculated. Basic assumptions for conducting logistic regression analysis, including independence of errors, i.e., lack of duplicate entries, linearity in the logit for continuous independent variables, absence of multicollinearity, and lack of strongly influential outliers, were tested and met. Linear relationships between continuous variables and the logit transformation of the dependent variable were tested with the Box–Tidwell test.

All regression analyses were conducted with a direct model-building approach, in which all independent variables were added simultaneously and having equal importance. Outcome (mortality/intensive care treatment/oxygen requirement) was set as the dichotomous dependent variable, while lipid parameters and predefined covariates were used as independent variables (method = enter).

We also provided a second measure of risk, i.e., hazard ratios, as calculated by a Cox proportional hazards model. The input here was analogous to the approach described in the previous paragraph, with the addition of a time parameter. Time to event was defined in days measured from hospital admission. Hazard ratios for in-hospital mortality were calculated with a 95% confidence interval.

To standardize the results and thus improve comparability between the risks associated with these lipid parameters, we conducted *z*-score normalization for each parameter. We then calculated the corresponding odds ratios and hazard ratios for the transformed parameters as described above. Odds ratios and hazard ratios were reported both for the continuous variables and the *z*-score-normalized variables to allow for direct comparison between these results.

We then adjusted the results from these models for potential confounding by the covariates age, type II diabetes, obesity, and statin therapy. To prevent overfitting of the regression models, we verified having a sufficient number of events in the whole cohort and the high-risk subgroups of diabetic and obese patients before adjusting for potential confounders. For better comparison, both unadjusted and adjusted odds ratios and hazard ratios are given.

In order to assess the relationship between anti-SARS-CoV-2 spike antibodies and lipid levels, parameters were stratified into categories of high and low. For lipid levels, groups were separated by the respective median value. For anti-SARS-CoV-2 antibody levels, the threshold was defined at 1200 U/mL [35]. We then combined anti-SARS-CoV-2 spike antibodies with each lipid parameter, forming the following groups: high/high, high/low, low/high, low/low.

Next, we calculated mortality rates and survival over time, first separately for each parameter, and second for the above mentioned combinations. Survival over time was then depicted using Kaplan–Meier graphs.

In order to test the robustness of our findings, we repeated the regression models described above while applying bootstrapping with 2000 samples, first for the whole cohort, second for diabetic patients, and third for obese patients. Finally, goodness of fit was tested using Hosmer–Lemeshow tests.

## 3. Results

### 3.1. Participants

**Figure 1:**
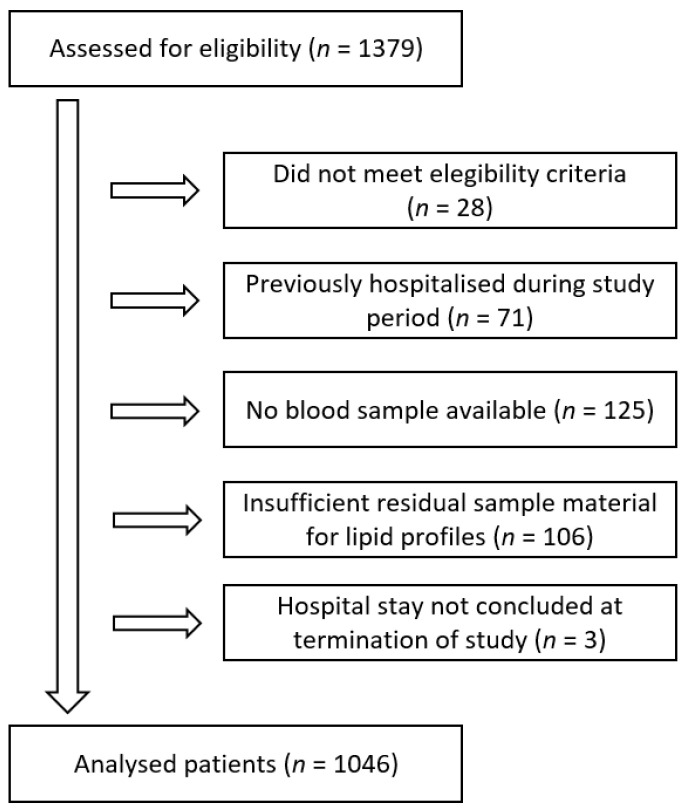
Patient flow diagram.

Overall, 1379 hospitalized patients were assessed for eligibility at five hospitals between 1 August 2021 and 10 April 2022. Of these, 1152 patients were originally enrolled in the study. However, due to insufficient residual sample material, lipid parameters could only be measured in 1046 patients. Patient flow is depicted in Figure 1.

In total, 118 patients died, 165 patients were admitted to an intensive care unit, and 587 patients required oxygen administration. Overall, 275 patients either had a previous diagnosis of type II diabetes or were diagnosed during their hospital stay, and 254 patients registered a BMI of 30 or above and were thus classified as obese. Patient characteristics are given in Table 1.

Patients with lower LDL-C or total cholesterol levels were significantly more likely to receive statin therapy and more likely to have a higher BMI and a history of diabetes and vascular disease.

### 3.2. Risk of Outcome by Lipid Levels

LDL-C, HDL-C, and total cholesterol levels were significantly lower in non-survivors than in survivors (mean LDL-C 56.1 mg/dL, 95%CI 50.4–61.8 vs. 72.6 mg/dL, 95%CI 70.2–75.0, *p* < 0.001; HDL-C 34.2, 95%CI 31.7–36.8 vs. 38.1 mg/dL, 95%CI 37.2–39.1, *p* = 0.025; total cholesterol 139.3 mg/dL, 95%CI 130.9–147.7 vs. 157.4, 95%CI 154.1–160.6, *p* = 0.002). Triglyceride concentrations were not significantly different between patients who survived and those who did not.

In order to determine odds ratios and thus quantify the respective risk associated with lower lipid levels, we first calculated odds ratios for the continuous lipid parameters by logistic regression analysis. We further provided hazard ratios as determined by a Cox proportional hazard model. Next, we calculated normalized *z*-scores for LDL-C, HDL-C, and cholesterol levels to standardize the results and improve comparability between the risks associated with these parameters. We then adjusted for potential confounders, including age, type II diabetes, and obesity.

Table 2 summarizes the estimated risks in the form of odds ratios and hazard ratios, stratified by lipid parameter both for continuous values and *z*-scores. Risk assessment for continuous variables is given per 10 mg/dL decrement since differences in lipid levels of ±1 mg/dL were not considered to be clinically relevant.

Risk of in-hospital mortality increased 1.7 times for each standard deviation decrease in LDL-C values, by 1.4 times for each decrease in HDL-C values, and by 1.5 times for each decrease in total cholesterol levels (aOR 1.73, 95%CI 1.30–2.31, *p* < 0.001, aOR 1.44, 95%CI 1.10–1.88, *p* = 0.008, aOR 1.49, 95%CI n1.14–1.94, *p* = 0.003).

In our Cox proportional hazard model, lower LDL-C, HDL-C, and total cholesterol levels were also associated with elevated risk of in-hospital mortality (aHR 1.15, 95%CI 1.08–1.23; *p* < 0.001, 1.18, 95%CI 1.10–1.26, *p* < 0.001; 1.14, 95%CI 1.07–1.22, *p* < 0.001). In this analysis, other covariates that showed a significant association with in-hospital mortality were age (*p* < 0.001) and statin therapy (*p* < 0.001), but not type II diabetes nor obesity. Standard deviations for each lipid parameter are included in Table 1.

Next, we divided lipid levels into high and low categories, separated by the respective median value, to assess the difference in mortality risk between high and low lipid levels. With regard to LDL-C, mortality risk increased two-fold for patients with LDL-C levels below 67 mg/dL (aOR 2.013, 1.250–3.241, *p* = 0.004; aHR 1.712, 1.102–2.661, *p* = 0.017). For TC and HDL-C levels, mortality risk did not differ significantly below vs. above the respective median value.

### 3.3. Risk of Outcome by Lipid Levels and Anti-SARS-CoV-2 Antibodies

Previous studies have suggested that lipoproteins are vital for the removal of pathogen-associated lipids and toxins in severe infections such as sepsis [1,2,3,4]. Similarly, antibodies are known to help neutralize and remove pathogens. With regard to COVID-19, anti-SARS-CoV-2 spike antibody levels have been shown to be inversely associated with outcome [35]. We therefore evaluated whether there was a relationship between antibody and lipoprotein levels and patient mortality.

**Figure 2:**
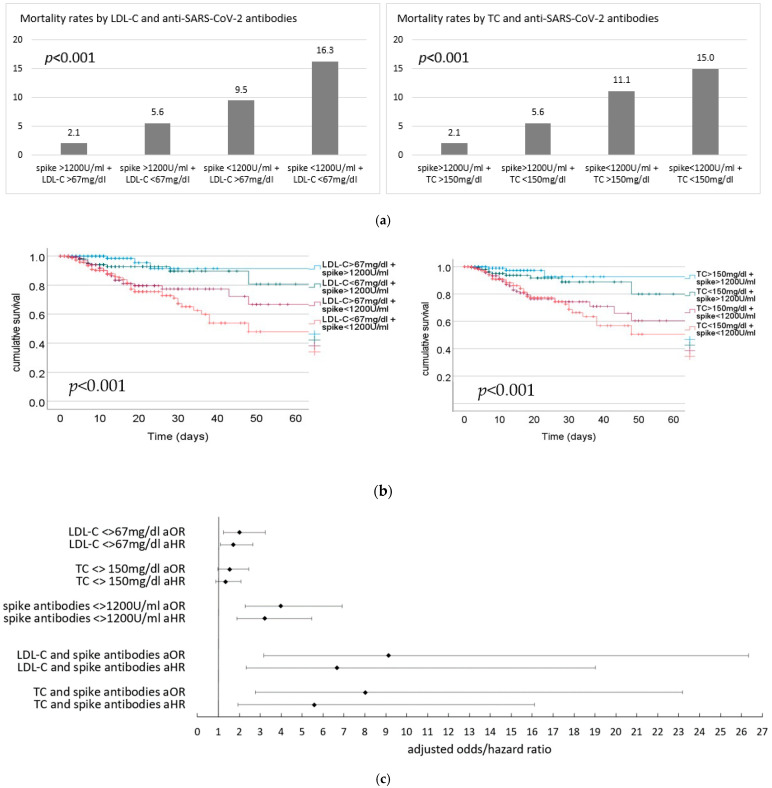
(**a**) Mortality rates (%) by LDL-C (**left**)/TC (**right**) and anti-SARS-CoV2-spike antibodies. TC total cholesterol. (**b**) Cumulative survival over time in days depending on lipid and anti-SARS-CoV2 spike antibody levels; LDL-C (**left**) and TC (**right**). TC total cholesterol. (**c**) Adjusted odds and hazard ratios for in-hospital mortality for lipid levels (below vs. above the respective median value), anti SARS-CoV2 antibodies (<>1200 U/mL) as well as aOR/aHR for low vs. high levels of both lipid level and anti-SARS-CoV2 spike antibodies. TC total choles-terol, spike antibodies—anti-SARS-CoV2-spike antibodies.

To this end, lipid levels and anti-SARS-CoV-2 spike antibodies were grouped into high and low categories. Lipid parameters and antibodies were then combined as follows—high/high, high/low, low/high, and high/high. Next, mortality rates and survival over time were calculated for all combinations. Results are depicted in Figure 2.

The lowest mortality rates occurred in patients with high levels of anti-SARS-CoV-2 antibodies and high levels of either LDL-C or total cholesterol. In contrast, patients with both low levels of LDL-C or total cholesterol and low levels of anti-SARS-CoV-2 antibodies showed the highest mortality rates.

For LDL-C, mortality rates varied between 2.1% for high levels of both LDL-C and anti-SARS-CoV-2 antibodies and 16.3% for low levels of LDL-C and anti-SARS-CoV-2 antibodies (aOR 9.137, 95%CI 3.169–26.343, *p* < 0.001; aHR 6.659, 95%CI 2.332–19.010, *p* < 0.001). Accordingly, for total cholesterol and anti-SARS-CoV-2 antibodies, mortality rates varied between 2.1% and 15.0% (aOR 8.012, 95%CI 2.769–23.181, *p* < 0.001; aHR 5.576, 95%CI 1.930–16.113, *p* = 0.002).

## 4. Discussion

In this prospective, multicenter cohort study on 1152 hospitalized patients with COVID-19, we are the first to show that lipid and anti-SARS-CoV-2 spike antibody levels are strongly associated with COVID-19 mortality.

Mortality rates were lowest in patients who had high levels of both LDL-C or TC and anti-SARS-CoV-2 spike antibodies. Conversely, patients who had low levels of both parameters registered the highest mortality rates.

Separately, plasma lipids and anti-SARS-CoV-2 antibodies have previously been shown to be associated with COVID-19 mortality. For instance, a systematic review and meta-analysis comprising more than 250.000 patients reported that severe COVID-19 patients had lower total cholesterol, HDL-C, and LDL-C levels than patients with non-severe courses [19]. A previous observational study from Spain described worse outcomes in hospitalized patients with low LDL-C and high triglyceride levels [13]. While we also observed worse outcomes in patients with low LDL-C levels, triglyceride levels did not differ significantly by outcome in our study population. This discrepancy may stem from differences in disease severity or fasting state at the time of sample collection. Our findings are in accordance with results from a meta-analysis that described lower total cholesterol, LDL-C, and HDL-C but not triglycerides in hospitalized patients with severe COVID-19 [20].

The pathophysiological role of lipoproteins in COVID-19 is still incompletely understood [5]. With regard to plasma lipid levels, it has previously been suggested that lipoproteins, especially HDL, play a crucial role in the removal of pathogen-associated lipids and toxins in severe infections such as sepsis [1,2,3,4]. Lower lipid levels in patients with COVID-19 may therefore indicate an impairment of this important biological process. Alternatively, higher lipid levels may simply be indicative of a better general condition of the individual, as low plasma lipid levels have been attributed to a hypermetabolic state and undernutrition in infected patients [2]. In addition to reduced levels of lipoproteins in COVID-19 patients, there is evidence to suggest that the function of lipoproteins may also be impaired. A study by Begue et al. reported that HDL particles from severely ill COVID-19 patients were less protective against permeability, VE-cadherin disorganization, and apoptosis than in healthy controls [22].

One of the main function of antibodies is the neutralization of pathogens. Previous studies have reported a positive correlation between neutralizing antibodies and protection against COVID-19 [23,24,25,26]. In addition, the administration of neutralizing monoclonal antibodies had positive effects in the prevention and treatment of COVID-19 [27,28,29]. Previous studies have demonstrated a protective effect of booster vaccinations against severe illness and hospitalization due to COVID-19 in the general population [30,31,32]. We previously reported that antibody levels on hospital admission are inversely related to in-hospital mortality in patients with COVID-19 [35].

Hypothetically, lipoproteins could be involved in the removal of SARS-CoV-2 particles that have previously been neutralized by antibodies. However, it is currently unclear if the association between lipoprotein and antibody levels and COVID-19 mortality indicates a pathophysiological mechanism or rather if they are representative of a better general condition that, in turn, improves chances of survival.

One of the major strengths of this study is its high recruitment rate, which reduces the risk of selection bias. Moreover, this investigation is based on a hard endpoint (in-hospital mortality) which is less susceptible to assessment bias because it does not require subjective clinical patient assessments [38]. In addition, we adjusted for multiple potential confounders, including statin therapy and known risk factors for COVID-19, including age, type II diabetes, and obesity [37,39].

With regard to limitations, this study was designed with a focus on hospitalized patients and its results therefore may not be applicable to outpatients. However, as severely ill patients are more likely to be treated in a hospital, we deemed it important to focus on this patient group. While we do not have follow-up data after discharge, this study was designed to examine the relationship between plasma lipoproteins, anti-SARS-CoV-2 antibodies, and mortality and thus centers on severely ill, hospitalized patients. Further, this study was conducted with residual sample material only; therefore, fasting periods could not be observed before sample collection, which may have affected triglyceride measurements in particular.

While we did see significant differences in outcome with decreasing concentrations of LDL-C, HDL-C, and total cholesterol, additional studies are required for determining a protective cutoff against negative outcomes in COVID-19. Our data suggest an association between lipoprotein and antibody levels and mortality in COVID-19. As both lipoproteins and antibodies have previously been associated with the removal of pathogens, a synergistic pathophysiological mechanism is theoretically possible. However, further studies are required to confirm such a relationship and to elucidate the exact mechanisms involved.

Understanding the combined role of lipoproteins and antibodies in severe infections, such as COVID-19, may help identify patients at risk, thereby allowing timely adjustment of therapy, and provide new options for future pharmacological therapeutic approaches.

In conclusion, our data suggest that LDL-C and TC-levels, combined with anti-SARS-CoV-2 spike antibodies, are a strong predictor of in-hospital mortality. Patients with low levels of LDL-C and total cholesterol combined with low levels of anti-SARS-CoV-2 antibodies exhibited the highest mortality rates.

## Figures and Tables

**Table 1:** Patient characteristics and outcomes by LDL-C and total cholesterol levels. Quantitative results are given as means ± standard deviation; lipid levels are given in mg/dL. BMI: body mass index, DM: diabetes mellitus, CAD: coronary artery disease, COPD: chronic obstructive pulmonary disease, TIA: transient ischemic attack, CVD: cerebrovascular disease, CT: cycle threshold, bold print: statistically significant.

	Whole Cohort *n* = 1152	LDL-C <67 mg/dL *n* = 525	LDL-C >67 mg/dL *n* = 520	*p*-Value	TC <150 mg/dL *n* = 520	TC >150 mg/dL *n* = 526	*p*-Value
age (years)	66.8 ± 20.3	67.7 ± 19.8	66.0 ± 19.9	0.116	68.2 ± 19.1	65.5 ± 20.5	**0.038**
male gender (%)	53.2	56.6	49.6	**0.024**	44.5	61.9	**<0.001**
BMI (kg/m^2^)	27.1 ± 6.5	27.7 ± 6.9	26.6 ± 5.8	**0.012**	27.4 ± 6.7	26.8 ± 6.1	0.135
DM (%)	23.9	30.5	17.3	**<0.001**	29.2	18.6	**<0.001**
hypertension (%)	50.5	55.0	45.8	**0.003**	56.0	44.9	**<0.001**
CAD (%)	21.6	25.5	16.3	**<0.001**	26.9	15.0	**<0.001**
heart failure (%)	7.2	8.0	6.0	0.196	7.7	6.3	0.368
COPD (%)	9.6	11.2	8.3	0.106	10.8	8.7	0.270
asthma (%)	2.4	2.3	2.7	0.673	2.3	2.7	0.713
renal disease (%)	22.9	27.7	18.6	**<0.001**	27.2	19.1	**0.002**
stroke/TIA/CVD (%)	11.7	15.2	8.8	**0.002**	16.5	7.6	**<0.001**
mortality (%)	10.2	12.6	6.7	**<0.001**	11.7	7.6	**0.024**
CT value	21.3 ± 6.6	21.3 ± 6.5	21.2 ± 6.5	0.652	21.5 ± 6.6	21.0 ± 6.5	0.224
total cholesterol	155.6 ± 50.3	120.2 ± 2.2	191.4 ± 42.3	**<0.001**	116.9 ± 22.7	193.9 ± 39.7	**<0.001**
LDL-C	71.0 ± 37.0	42.7 ± 15.6	99.5 ± 29.6	**<0.001**	44.9 ± 18.1	96.8 ± 32.4	**<0.001**
HDL-C	37.7 ± 14.8	34.2 ± 13.2	41.4 ± 15.4	**<0.001**	32.8 ± 11.3	42.7 ± 16.1	**<0.001**
triglycerides	139.8 ± 83.8	132.7 ± 80.6	146.9 ± 86.5	**<0.001**	119.0 ± 51.7	160.3 ± 103	**<0.001**
statin therapy (%)	25.7	36.0	16.5	**<0.001**	35.4	17.3	**<0.001**

**Table 2:** Odds ratios and adjusted odds ratios for each lipid parameter both for the continuous variable in decrements of 10 mg/dL (left) and for the z-scores of each variable (right). Adjusted odds ratios were adjusted for age, obesity, diabetes type II, and statin therapy. OR: odds ratio, aOR: adjusted odds ratio, HR: hazard ratio, ICU: intensive care unit, bold print—statistically significant.

	OR, 95%CI	*p*-Value	aOR, 95%CI	*p*-Value	OR z-Scores, 95%CI	*p*-Value	aOR z-Scores, 95%CI	*p*-Value
**LDL-C**					**LDL-C**			
mortality	1.16, 1.08–1.24	**<0.001**	1.16, 1.07–1.25	**<0.001**	1.73, 1.34–2.23	**<0.001**	1.73, 1.30–2.31	**<0.001**
mortality, HR	1.04, 1.02–1.06	**<0.001**	1.04, 1.02–1.06	**<0.001**	1.16, 1.09–1.23	**<0.001**	1.15, 1.08–1.23	**<0.001**
**HDL-C**					**HDL-C**			
mortality	1.23, 1.05–1.44	**0.012**	1.28, 1.07–1.53	**0.008**	1.35, 1.07–1.71	**0.012**	1.44, 1.10–1.88	**0.008**
mortality, HR	1.11, 1.06–1.16	**<0.001**	1.12, 1.07–1.17	**<0.001**	1.16, 1.09–1.24	**<0.001**	1.18, 1.10–1.26	**<0.001**
**total cholesterol**					**total cholesterol**			
mortality	1.09, 1.04–1.14	**<0.001**	1.08, 1.03–1.14	**0.003**	1.51, 1.20–1.92	**<0.001**	1.49, 1.14–1.94	**0.003**
mortality, HR	1.03, 1.02–1.04	**<0.001**	1.03, 1.01–1.04	**<0.001**	1.15, 1.09–1.23	**<0.001**	1.14, 1.07–1.22	**<0.001**

## Data Availability

As personal individual information is included in the dataset, the data pertaining to this investigation is not publicly available to protect study participant privacy. However, an anonymised version will be shared upon reasonable request to the corresponding author.

## Abbreviations

COVID-19	coronavirus disease 2019
SARS-CoV-2	severe acute respiratory syndrome coronavirus 2
PCR	polymerase chain reaction
ICU	intensive care unit
OR, aOR, HR	odds ratio, adjusted odds ratio, hazard ratio
CI	confidence interval
LDL-C	low density lipoprotein
HDL-C	high density lipoprotein
BMI	body mass index
COPD	chronic obstructive pulmonary disease
CAD	coronary artery disease
TIA	transient ischemic attack
CVD	cerebrovascular disease
